# Sickle Cell Disease (SCD) Leading to Pulmonary Arterial Hypertension (PAH) and Cholelithiasis (CL)

**DOI:** 10.7759/cureus.37113

**Published:** 2023-04-04

**Authors:** Saran Chacko, Ulhas Jadhav, Babaji Ghewade, Pankaj Wagh, Roshan Prasad, Mayur B Wanjari

**Affiliations:** 1 Respiratory Medicine, Jawaharlal Nehru Medical College, Datta Meghe Institute of Higher Education and Research, Wardha, IND; 2 Community Medicine, Jawaharlal Nehru Medical College, Datta Meghe Institute of Higher Education and Research, Wardha, IND; 3 Research and Development, Jawaharlal Nehru Medical College, Datta Meghe Institute of Higher Education and Research, Wardha, IND

**Keywords:** gall stones, anemia, cholelithiasis, pulmonary arterial hypertension, sickle cell disease

## Abstract

Sickle cell disease (SCD) consists of a variety of hereditary hemoglobinopathies linked to alterations in the beta component of the hemoglobin (Hb) molecule. Acute SCD manifestations include stroke, acute chest syndrome (ACS), and pain, whereas chronic manifestations include avascular necrosis, chronic renal disease, and gallstones. This case report describes a rare instance of SCD-related pulmonary arterial hypertension (PAH) and cholelithiasis (CL). Following investigations, such as high-resolution CT scan thorax, chest X-ray, two-dimensional echocardiography, and ultrasonography of the abdomen and pelvis, PAH and CL were confirmed. The medical intervention mainly involved oxygenation, IV fluids, IV antibiotics, simple packed red blood cell transfusion (SBCT), folic acid, calcium supplementation, hydroxyurea, chest physiotherapy, and respiratory muscle strengthening exercises. The surgical intervention for CL was planned. Hence, the learning point from this case is that early multidisciplinary approach should be taken in order to control the progression of SCD.

## Introduction

Sickle cell disease (SCD) is a diverse set of hereditary hemoglobinopathies linked to alterations in the beta component of the hemoglobin (Hb) molecule. Since it is an autosomal recessive disease, disease states and complications would only be present in individuals who were homozygous for the mutant allele or who were found to be compound heterozygotes with mutant alleles linked to other hemoglobinopathies (such as HbC disease, sickle cell Hb/beta-thalassemia, etc) [1]. For diagnosing SCD , certain Hb tests like Hb electrophoresis and sickling test are needed. Recurrent vaso-occlusive pain crises are a characteristic of SCD, regardless of its clinical symptoms, and any organ could be permanently damaged by these persistent ischemic injuries. Chronic hemolysis, which causes considerable bilirubin generation, can lead to gallstone formation. One of the key symptoms of the condition is pigment gallstone development, which is caused by the bilirubin generated [1]. Gallstone development in SCD patients may also be influenced by changes in the function of the gall bladder or bile acid metabolism.

One of the most significant gastrointestinal signs of SCD is the development of gallstone [1,2]. which is mostly brought on by too much cholesterol in bile. Gallstones, which can lead to gall bladder cancer, are the main cause of death in underdeveloped nations. It is estimated to be around 4% in India compared to 10% in the West [3]. Gallstones can develop in SCD patients at different ages: 15% before the age of 10, 22% between the ages of 10 and 14, 36% between the ages of 15 and 18, and 70% by the time the patient is over 30 [4]. Pigment gallstones are more common in people with sickle cell anemia (SCA) and thalassemia. Increased excretion of unconjugated bilirubin, bilirubin precipitation, and crystallization of bilirubin are the three factors that lead to the development of gallstones. Cholelithiasis (CL) presents with a variety of signs and symptoms, such as extreme pain at Murphy's point in the right upper abdominal quadrant (UAQ), bilious vomiting, a mild to moderate rise in temperature, obstructive jaundice, lack of appetite, and weight loss. It begins in childhood at a young age because of various underlying etiological factors for SCD. A study by Arvind Kumar et al. found that mixed gallstones are more common in females [5].

Another complication of SCD is pulmonary arterial hypertension (PAH). Understanding the role of SCD in the pathogenesis, categorization and prognosis of the PAH is important. According to the European Society of Cardiology (ESC) guidelines, mean pulmonary artery pressure (mPAP) above 20 mm of mercury is defined as PAH, which occurs in 6-11% of SCD individuals. The ESC and the European Respiratory Society's Task Force for the Diagnosis and Treatment of PAH includes SCD-associated PAH and other hemolytic anemias in Group 1. Therefore, this case report aimed to highlight the effectiveness of medical intervention in this case of SCD-caused PAH and CL.

## Case presentation

A 38-year-old male presented with the chief complaints of right UAQ pain, with sporadic bouts of persistent, sharp pain, bilious vomiting, and nausea, accompanied by generalized weakness, which lasted for a month, loss of appetite and weight, along with productive cough and dyspnea, both of which lasted for seven days. Additionally, the patient had a family history of SCD as well as a history of a pleural effusion associated with tuberculosis 12 years previously. On examination, he had abdominal distension with tenderness in right UAQ, rebound tenderness, a palpable abdominal mass in right UAQ, anasarca, and jaundice. The vital signs included a temperature of 102°F, tachycardia (102 beats per minute), respiratory rate (19 breaths per minute), and peripheral capillary oxygen saturation (SpO_2_) of 88%. Based on the presentation and findings, his blood sample was sent for complete blood count (CBC), which illustrated Hb levels of 7g/dl, increased white blood cell of 13,000 and reticulocyte count more than 3 per cent, along with positive sickling test, whereas liver function test reports revealed decreased alkaline phosphatase levels of 30 IU and increased total bilirubin levels of 4.5gm/dl that resulted in unconjugated hyperbilirubinemia. The data are summarized in Table1.

**Table 1:** Summary of laboratory investigations Hb: Hemoglobin

INVESTIGATIONS	RESULTS
Hb	7 gram% (14-17 gram%)
Total leucocytic count	13,000 cells per mm^3^ (4,000-10,000 cells per mm^3^)
Reticulocytic count	5% (0.5-2.5%)
Total bilirubin	4.5 mg/dl (upto 1.2 mg/dl)
Conjugated bilirubin	0.8 mg/dl (less than 0.3 mg/dl)
Unconjugated bilirubin	3.7 mg/dl (0.2-0.8 mg/dl)
Sickling test	Positive

**Figure 1:**
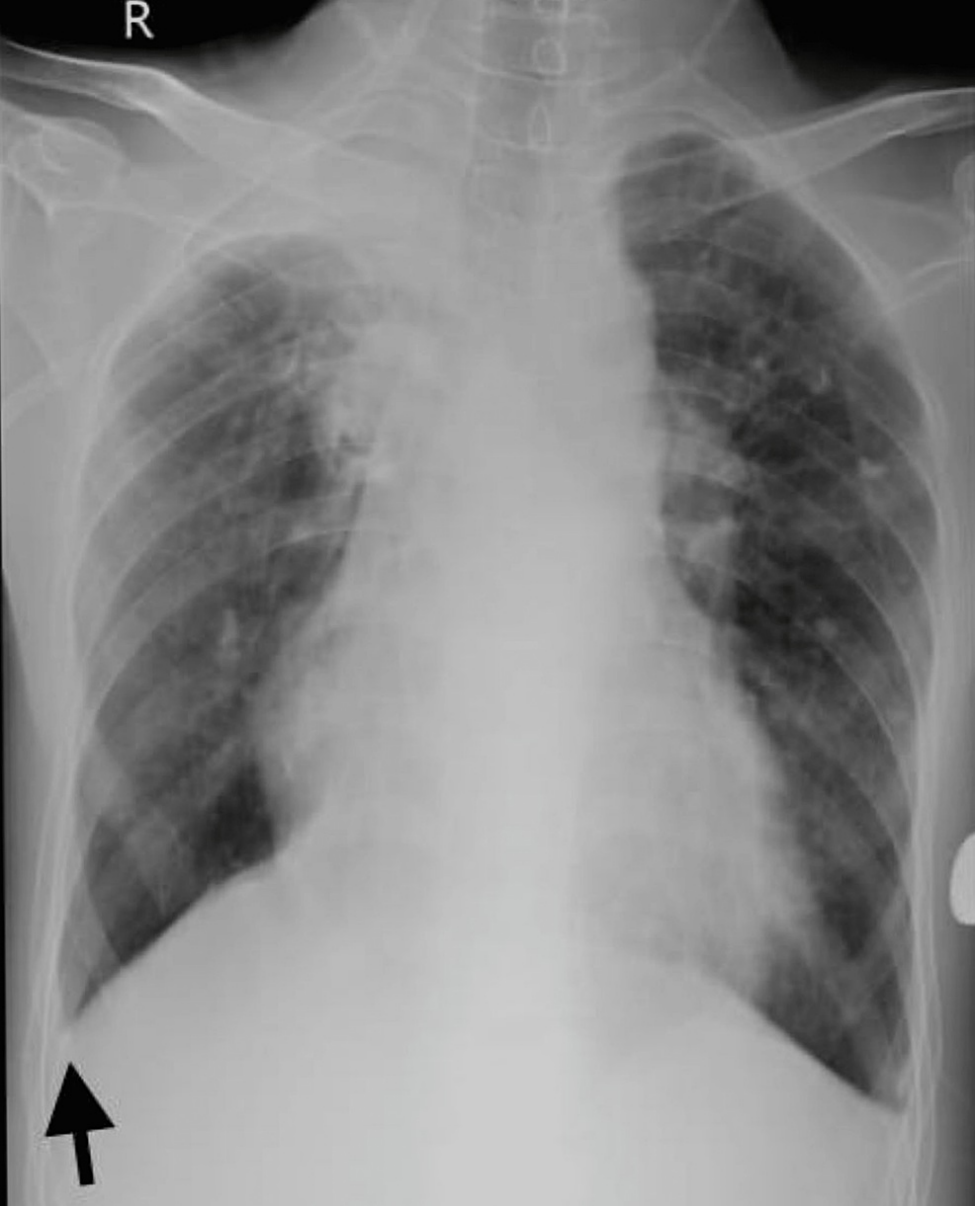
Chest X-ray in postero-anterior view; the arrow shows blunting of right costophrenic angle, which is suggestive of pleural effusion

As the patient had a history of tubercular pleural effusion 12 years ago, sputum examination for acid fast bacilli was done to rule out pulmonary tuberculosis. Diagnostic assessment mainly involved ultrasonography of the abdomen and pelvis that showed collapsed gall bladder with hyperechoic focus noted gallstones of 22mm and 28mm, with the post-acoustic enhancement that confirmed CL as well as auto-splenectomy as spleen was not visualized. Additionally, two-dimensional echocardiography demonstrated dilated right-side atrium and ventricle, mPAP of 29mmHg with mild tricuspid regurgitation having a jet velocity of 2.5m/s along with jugular venous pressure at 4cm above sternal angle indicating moderate PAH. Furthermore, chest X-ray was also recommended (Figure 1).

**Figure 2:**
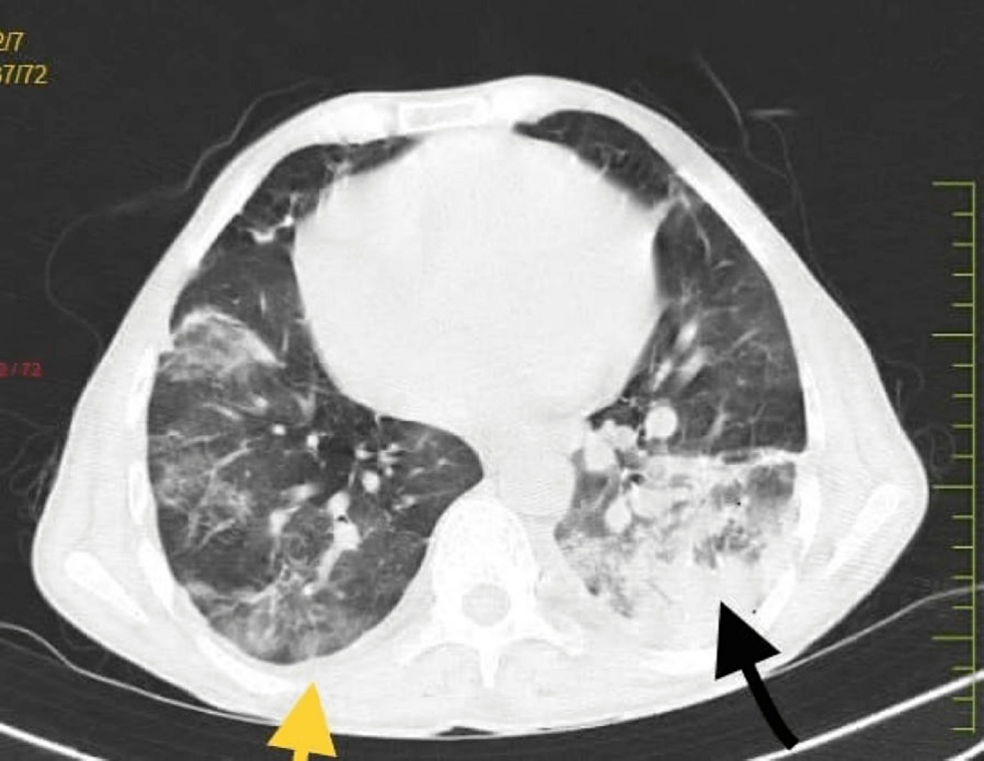
High-resolution CT scan of chest: the yellow arrow shows small right-sided effusion and the black arrow shows left lower lobe consolidation.

The patient also underwent a CT scan (Figure 2).

The results indicated multifocal pulmonary infiltrates with right-side pleural effusion and upper lobe consolidation along with altered thoracic spine and cardiomegaly. Referral, treatment, and follow-up of the patient were managed by an interdisciplinary team that included a consultant hematologist, anesthesiologist, pulmonologist, and surgeons with laparoscopic experience. Upon admission, adequate oxygenation was provided to correct hypoxia along with IV hydration. A straightforward packed red blood cell transfusion (SBCT) was used to increase Hb levels to 10g/dl, along with the administration of hydroxyurea to increase fetal Hb and decrease sickling and anticoagulation therapy to prevent blood clots [4]. Additionally, folic acid and calcium supplements were given. Sildenafil, which facilitates easy blood flow by relaxing the blood vessels in the lungs, along with IV antibiotics and dexamethasone 4mg was given. As the spleen was not visualized due to auto-splenectomy, the patient was advised to continue these medications lifelong for the purpose of immunization. The treatment was also assisted with nebulization with bronchodilators and steroids, along with physiotherapeutic intervention that mainly included chest physiotherapy, incentive spirometry, and early mobilization to prevent atelectasis and bedrest complications.The patient adhered well to the given treatment, following which he was symptomatic for CL. After taking consultation from surgeons, laparoscopic cholecystectomy was planned.

## Discussion

This report highlights a rare case of CL combined with PAH caused mainly due to SCD and the impact of medical intervention on the patient. A study by Arvind Kumar et al. showed that mixed gallstones more common among females [5]. The prevalence of gallstones in SCD is much higher than in the normal population, and more in males than in females [6]. A remarkable clinical feature of sickle-related pulmonary hypertension (PH) is its high mortality despite relatively low mPAPs [7]. It is crucial to take into account the above-mentioned domains in the future recommendations to provide high-risk individuals with optimal and individualized care and to decrease the advancement of the disease, morbidity, and death rates in light of the prevalence of PAH in patients with SCD. SBCT was performed to get Hb levels above 10g/dl along with anticoagulation therapy to prevent blood clots, and administration of hydroxyurea as it increases fetal Hb and reduces sickling which corresponds well with previous studies. An increased risk for mortality is defined as a tricuspid regurgitant velocity (TRV) equal to or greater than 2.5 m/second, an NT-pro-BNP level equal to or greater than 160pg/ml, or right heart cathetrization (RHC) confirmed PH [8].

The main vasculopathic complication of SCD seen in this case is PAH, for which the patient was given sildenafil tablet, which is a beneficial drug in relieving symptoms, is easy to use, has low cost, has a basic dosing regimen, and provides a greater quality of life. Sildenafil can be a suitable option in the treatment of patients with PAH as it corresponds well with review literature given by Bhogal et al. [9]. For patients diagnosed with PAH by right-heart catheterization, referral for clinical trials is recommended; PH-specific therapy can be considered judiciously in the absence of definitive clinical trials outcome data [10]. The only medication that has been successfully demonstrated in prior studies to lower the frequency of painful episodes, rate of acute chest syndrome (ACS), and blood transfusions by 50% in adults is hydroxyurea, which is a myelosuppressive agent. It raises the levels of fetal Hb and Hb and was first tested in SCD in 1984 [11,12]. This group of patients should be informed about the symptoms of CL, its risk, and related comorbidities, as well as the necessity for routine preventative measures and regular follow-up because the incidence of CL in SCD is age-dependent. In our case, the cholecystectomy was planned. Along with the above-mentioned interventions adequate oxygenation, IV hydration, analgesics, and chest physiotherapy assisted the treatment, which correlates well in accordance with previous studies that add quality-adjusted life years and provide a stronger message towards lowering subsequent hospital expenses. However, all of these factors call for additional research that focuses on certain treatment strategies, combines specialized approaches, and perhaps even anticipates the effects of these variations in the incidence of mortality and morbidity.

## Conclusions

This report highlights a rare case of CL and PAH as result of SCD and observes the impact of the interventions performed in this case. Since the treatment in such a condition necessitates an interdisciplinary care team. This case study concludes that the patient adhering effectively to the medical interventions interrupts the progression of the vicious cycle of disease worsening. Additionally, early screening with laboratory tests and echocardiography should be taken into consideration to start intensive treatment to prevent disease progression and lower mortality rates.
